# Transperineal versus transrectal systematic prostate biopsy in routine clinical practice: a real-world comparative study

**DOI:** 10.3389/fruro.2026.1806961

**Published:** 2026-03-19

**Authors:** Anouar El Ghazzaly, Mohammed Mrabeti, Abdesamad El Bahri, Larbi Hamedoun, Mohammed Tetou, Mohammed Alami, Ahmed Ameur

**Affiliations:** 1 Department of Urology, Mohamad V Military Teaching Hospital, Rabat, Morocco; 2 Faculty of Medicine and Pharmacy, Mohammed V University in Rabat, Rabat, Morocco

**Keywords:** biopsy, cancer, diagnosis, prostate, transperineal, transrectal

## Abstract

**Purpose:**

Prostate biopsy is the reference standard for confirming prostate cancer in men with clinical suspicion. The transrectal (TR) route is widely used but carries a risk of infectious complications and may sample anterior regions less effectively. Transperineal (TP) biopsy has emerged as a safer alternative with lower infectious risk. However, real-world comparative evidence between the routes is limited in some settings. The objective is to compare the diagnostic yield, tissue quality, and complications of systematic TR versus TP prostate biopsy in a Moroccan tertiary center.

**Methods:**

In this retrospective study, 139 men with suspected prostate cancer underwent systematic biopsy via TR or TP biopsy. Analyses were restricted to systematic cores. Biopsy quality was assessed by median core length. Complications were graded using the Clavien–Dindo classification.

**Results:**

A total of 139 men underwent systematic biopsy. Baseline clinical characteristics were similar across most variables, except DRE, between groups. TP yielded a longer median core length than TR (p = 0.02). In contrast, detection rates of clinically significant prostate cancer (csPCa) were similar (40.3% for TR vs 38.9% for TP). Several clinical factors were associated with csPCa detection, including higher PSA, higher PSA density, suspicious DRE, and higher PI-RADS category. In contrast, anterior lesion location was associated with a lower risk of csPCa. Regarding complications, infectious complications were more common after TR biopsy (7.5% compared to 1.4%). Additionally, acute urinary retention was seen in 6.0% of TR cases and 8.3% of TP cases. No Clavien–Dindo grade III or higher events were reported.

**Conclusion:**

TP systematic biopsy provides csPCa detection comparable to TR biopsy, yields longer cores, and shows a lower observed rate of infectious complications, supporting TP adoption to reduce infectious morbidity without compromising diagnostic performance.

## Introduction

Prostate cancer (PCa) is a major global health issue, ranking as the second most common cancer and the fifth leading cause of cancer death in men (1). An aging population and wider PSA testing are increasing the demand for diagnostic evaluation (2). Despite advances in non-invasive tools such as multiparametric MRI (mpMRI) and improved PSA-based risk stratification, histological confirmation by biopsy remains essential, and systematic biopsy is still relevant in men with elevated PSA and normal or negative mpMRI findings (3). Transrectal (TR) biopsy has historically been the most widely used approach because it is simple and broadly available, but it is limited by a higher risk of infectious complications. In contrast, the transperineal (TP) approach avoids the rectal mucosa and is associated with fewer infectious complications (4). Numerous studies report similar detection rates of clinically significant prostate cancer (csPCa) between TP and TR biopsies, with an overall better safety profile for TP (4–8), leading to recommendations advocating TP as the preferred approach (9).

However, evidence from different practice settings remains limited. Therefore, we did a retrospective study using routine clinical data at a Moroccan university referral center to compare the diagnostic yield, biopsy quality, and complications of TP and TR systematic prostate biopsies.

## Methods

### Study design

This was a retrospective, single-center, observational cohort study conducted at the Department of Urology, Mohammed V Military Teaching Hospital, Rabat, Morocco. The study aimed to compare the diagnostic performance, biopsy quality, and complication rates of systematic transrectal (TR) versus transperineal (TP) prostate biopsy in routine clinical practice.

The study period reflected an institutional transition in biopsy technique. Patients who underwent transrectal biopsy between January 2023 and June 2023 formed the TR cohort, while those biopsied using the transperineal approach between July 2023 and January 2024 constituted the TP cohort. No overlap occurred between cohorts.

The study was approved by the local institutional ethics committee and was conducted in accordance with the Declaration of Helsinki.

### Study population

Consecutive adult male patients (≥18 years) referred for prostate biopsy due to clinical suspicion of prostate cancer were eligible for inclusion. The indications for biopsy included a PSA of 4.0 ng/mL or higher, a DRE that had signs of abnormality, and/or imaging findings from mpMRI that were suspicious (PI-RADS 3 or above).

Patients were excluded if they had been previously diagnosed with prostate cancer, had evidence of an active urinary tract infection at the time of biopsy, had medical contraindications that prohibited them from having a biopsy, or if clinical or pathological results were missing or incomplete for any reason.

All patients provided written informed consent prior to undergoing the biopsy procedure.

Of the total of 139 consecutive patients, 67 had transrectal biopsy and 72 had transperineal biopsy.

### Pre-biopsy assessment

Age, prostate volume, PSA density (PSAD), DRE findings, and mpMRI results when available were among the baseline demographic and clinical data gathered.

Based on transrectal ultrasound data, the ellipsoid formula was used to estimate prostate volume (10). Serum PSA divided by prostate volume was used to define PSA density. Normal or suspect results of digital rectal inspection were classified according to the presence of nodularity or induration.

Experienced radiologists examined multiparametric MRI results following Prostate Imaging–Reporting and Data System (PI-RADS) version 2.1. PI-RADS category and anatomical location (posterior vs anterior prostate) define lesions.

### Biopsy procedures

All biopsies were performed by senior urologists experienced in prostate biopsy techniques and trained in standardized protocols.

#### Antibiotic prophylaxis and preparation

Before the operation, all patients were given a 400 mg oral dose of cefixime and a rectal enema. Before biopsy, patients were clinically evaluated for indications of infection.

#### Transrectal biopsy technique

Transrectal biopsies were performed with patients in the left lateral decubitus position using a transrectal convex ultrasound probe. Local anesthesia was administered via periprostatic nerve block. Under real-time ultrasound guidance, a systematic 12-core biopsy scheme was used, sampling standard sextant regions bilaterally with an 18-gauge biopsy needle.

#### Transperineal biopsy technique

Transperineal biopsies were performed under spinal anesthesia with patients in the lithotomy position. A side-firing transrectal ultrasound probe was used for imaging guidance. A freehand transperineal technique without a template was employed, allowing systematic sampling through the perineal skin. Twelve cores were obtained using an 18-gauge biopsy needle, targeting predefined anatomical regions in a standardized manner.

For patients with mpMRI lesions classified as PI-RADS ≥ 3, additional targeted biopsies were performed according to institutional practice. However, to ensure comparability between techniques, only systematic biopsy cores were included in the final analysis.

### Pathological evaluation and definitions

Under predetermined procedures, specialized uropathologists processed and examined all biopsy specimens. For every biopsy sample, core length was measured; biopsy quality was judged using the median core length per patient.

The International Society of Urological Pathology (ISUP) Grade Group system was reported in terms of prostate cancer grading (11). Clinically significant prostate cancer (csPCa) was defined as ISUP Grade Group ≥ 2.

### Outcome measures

The primary outcome was the detection rate of clinically significant prostate cancer based on systematic biopsy cores.

Secondary outcomes included: detection rate for prostate cancer overall; median core length to provide assessment of biopsy quality; and rates of complications within thirty days following biopsy.

Follow-up visits recorded complications using the Clavien–Dindo classification scheme (12). Among infectious issues were acute prostatitis and febrile urinary tract infections. Other complications evaluated included acute urinary retention and bleeding events (hematuria, hemospermia, rectorrhagia).

### Statistical analysis

Continuous variables were described as mean ± standard deviation or median with inter-quartile range based on the distribution of the variable. Categorical variables were reported as the number of patients and percentage of patients for each variable. To perform between-group comparisons, Student’s t-test or Mann–Whitney U test was used for continuous variables and Chi-square test or Fisher’s exact test was used for categorical variables. Univariate logistic regression analysis was utilized to assess the crude association between clinical variables and csPCa detection. In addition, a multivariable logistic regression model was constructed to evaluate the independent association between biopsy route and csPCa detection, adjusting for PSA, prostate volume, digital rectal examination status, and PI-RADS category. Given sparse data in low PI-RADS scores, PI-RADS was grouped as ≤3, 4, and 5 to ensure model stability. To further assess the robustness of results, subgroup analyses were performed on selected clinical characteristics to determine whether the impact of biopsy route differed across patient subgroups. Statistical significance was defined as a two-tailed p-value <0.05. All analyses were performed in R (version 4.5.2) using RStudio (version 2025.09.2 + 418).

## Results

### Patient demographics and baseline clinical characteristics

The study enrolled 139 adult men and treated them with either a transrectal (TR) biopsy (67 men) or a transperineal (TP) biopsy (72 men). **Table 1** compares the baseline demographic information of the two groups and shows that the groups were similar. Both groups had similar distributions of the Prostate Imaging-Reporting and Data System (PI-RADS) and locations of lesions identified by multi-parametric magnetic resonance imaging (mpMRI). There was a statistically significant difference in the median biopsy quality of the two groups, with the TP group producing longer median cores than the TR group (11.4 mm vs 10.2 mm; p = 0.02). Regarding digital rectal examination (DRE), there was a higher proportion of DRE findings classified as suspicious in the TR group than in the TP group (36% vs 17%; p = 0.010).

**Table 1:** Baseline characteristics of the study population.

Characteristic	TR (N = 67)	TP (N = 72)	p-value
Age (years)	67 (61, 71)	67 (63, 70)	0.9
PSA (ng/mL)	13 (8, 21)	11 (8, 17)	0.5
Prostate volume (mL)	50 (35, 60)	44 (31, 69)	0.6
PSA Density (ng/mL²)	0.23 (0.15, 0.41)	0.22 (0.15, 0.41)	0.9
DRE Findings			0.01
Normal	43 (64%)	60 (83%)	
Suspicious	24 (36%)	12 (17%)	
PI-RADS Category			0.4
PI-RADS ≤ 2	5 (7.5%)	2 (2.8%)	
PI-RADS 3	6 (9.0%)	7 (9.7%)	
PI-RADS 4	31 (46%)	42 (58%)	
PI-RADS 5	25 (37%)	21 (29%)	
Lesion Location			0.4
Posterior	45 (67%)	43 (60%)	
Anterior	22 (33%)	29 (40%)	
Longer median cores (mm)	10.2(9.4,10.8)	11.4 (10.7, 12.00)	0.02

Median [Q1, Q3]; n (%).

Wilcoxon rank sum test; Pearson’s Chi-squared test; Fisher’s exact test.

### Cancer detection outcomes

Clinically significant prostate cancer was detected in 27/67 patients (40.3%; 95% CI, 28.5–53.0) in the transrectal biopsy group and in 28/72 patients (38.9%; 95% CI, 27.6–51.1) in the transperineal biopsy group.

**Table 2** shows the impact of baseline characteristics on csPCa detection. The odds of detecting csPCa after transperineal biopsy compared to transrectal biopsy are 0.94 (95% CI 0.48-1.86; p = 0.865).

**Table 2:** Effect of baseline variables on the detection of clinically significant prostate cancer detection.

Variable	OR	95% CI	p-value
Biopsy route (TP vs TR)	0.94	0.48 - 1.86	0.865
Age (per 10 years)	1.59	0.97 - 2.71	0.067
PSA (per 1 ng/mL)	1.06	1.02 - 1.10	0.002
Prostate volume (per 1 mL)	0.99	0.98 - 1.00	0.082
PSA density (per 0.1)	1.38	1.17 - 1.69	0.001
DRE (suspect vs non)	2.43	1.13 - 5.33	0.024
PI-RADS			0.040
PI-RADS 3	1.80	0.18 - 41.1	
PI-RADS 4	3.32	0.53 - 64.5	
PI-RADS 5	7.14	1.10 - 141	
Lesion Location (Anterior vs Posterior)	0.28	0.12 - 0.59	0.001

Other clinical factors were identified as predictors of csPCa, including PSA level (OR 1.06; p = 0.002) and PSA density (OR 1.38; p < 0.001), which showed a positive association with the diagnosis of csPCa. However, for men whose DRE raised suspicion of csPCa, there was a greater than twofold increase in being diagnosed with csPCa (OR 2.43; 95% CI 1.13–5.33; p = 0.024).

Regarding the MRI results, a statistically significant correlation was found between the PI-RADS score and csPCa (p = 0.040), with the PI-RADS 5 score having the highest odds ratio (OR) (7.14) compared to PI-RADS ≤ 2.

The presence of an anterior lesion decreased the likelihood of detecting csPCa in this patient cohort (OR = 0.28; 95% CI: 0.12–0.59; p = 0.001). Age and prostate volume did not show statistically significant differences. Core length was significantly greater with the TP approach than with TR (11.4 mm [10.8–12.0] vs 10.2 mm [9.4–10.7], *p* = 0.02), suggesting a higher tissue yield with TP.

In multivariable logistic regression adjusting for PSA, prostate volume, digital rectal examination status, and grouped PI-RADS category (≤3, 4, and 5), biopsy route was not independently associated with csPCa detection (**Table 3**). Compared with the transrectal approach, the transperineal approach showed no significant difference in the odds of detecting csPCa. Higher PSA was independently associated with increased csPCa detection, whereas prostate volume showed a non-significant trend toward lower csPCa detection. DRE status and PI-RADS category were not statistically significant predictors in the adjusted model.

**Table 3:** Multivariable logistic regression for csPCa detection.

Variable	aOR	95% CI	p-value
Biopsy route (TP vs TR)	1.11	0.52–2.35	0.789
PSA (per 1 ng/mL)	1.05	1.00–1.11	0.033
Prostate volume (per 1 mL)	0.99	0.98–1.00	0.066
Suspicious DRE (yes vs no)	1.49	0.61–3.66	0.386
PI-RADS 4 (vs ≤3)	1.83	0.53–6.30	0.340
PI-RADS 5 (vs ≤3)	2.93	0.80–10.73	0.104

Multivariable logistic regression assessing the association between biopsy route (TP vs TR) and csPCa detection, adjusted for PSA, prostate volume, DRE (suspicious vs non-suspicious), and grouped PI-RADS category (≤3, 4, 5). Results are reported as adjusted odds ratios (aOR) with 95% confidence intervals.

**Figure 1:**
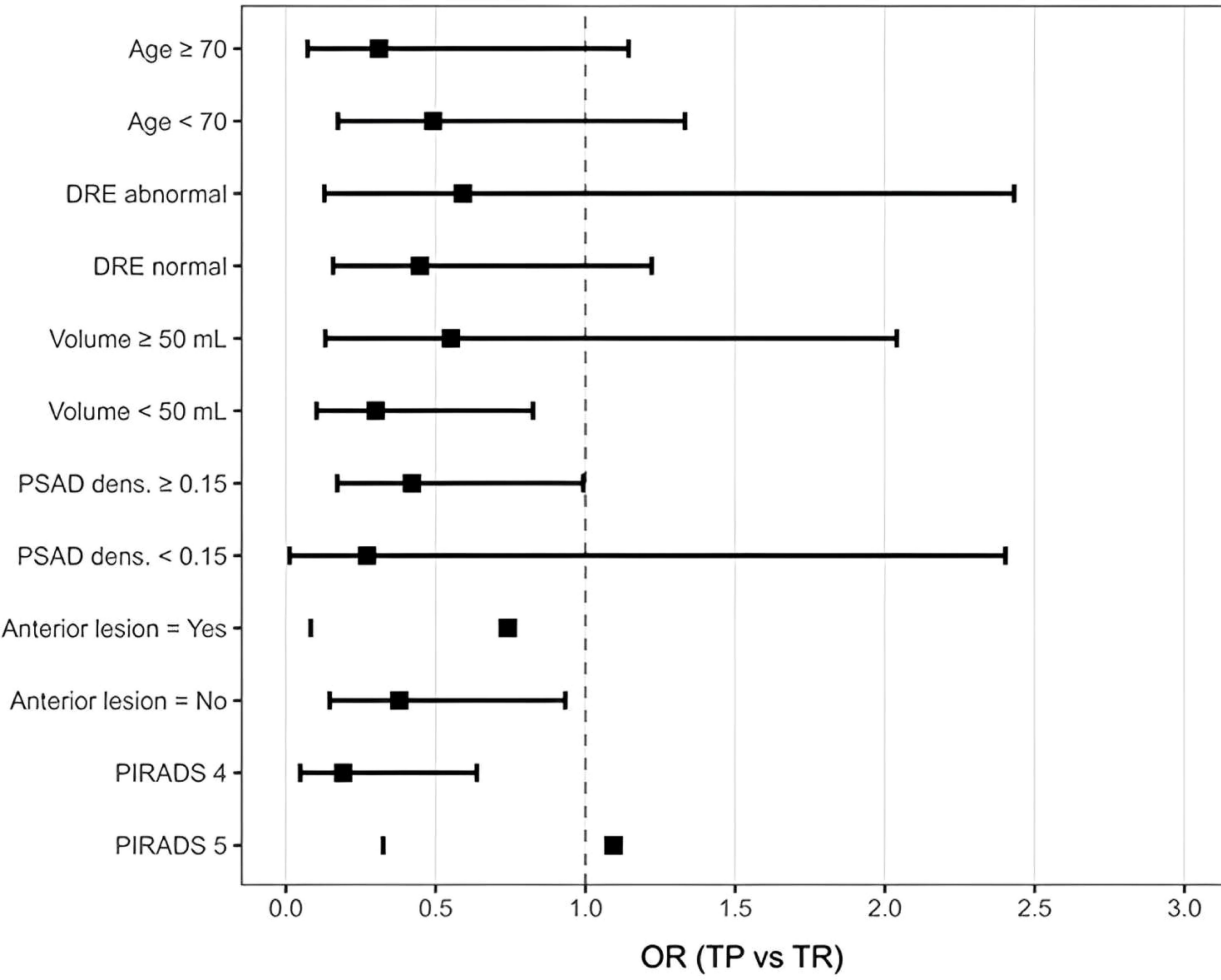
Forest plot showing odds ratios with 95% confidence intervals for the detection of clinically significant prostate cancer across patient subgroups.

The analysis of subgroups of men who underwent transperineal or transrectal biopsy is shown in **Figure 1**, and the confidence intervals for the odds ratios of each subgroup include the value 1.0.

### Complication outcomes

Complication rates according to the Clavien–Dindo classification system are shown in **Table 4**. The transperineal approach was associated with a lower observed rate of infectious complications than the Transrectal approach, but due to the size of the study, there was no statistically significant difference in this aspect of complication rate. The TR group had a higher observed rate (7.5%) with an infectious complication (febrile UTI or prostatitis) than the Transperineal (TP) group (1.4%). Although not statistically significant (p=0.11), the clinical outcome suggests a lower rate of infectious complications.

**Table 4:** Complications within 30 days after biopsy by route.

Complication type	TR	TP	p-value
Infectious Complications (Febrile UTI/Prostatitis)	5 (7.5%)	1 (1.4%)	0.11
Bleeding Events (hematuria, hemospermia or rectorrhagia)	3 (4.5%)	1 (1.4%)	0.4
Acute urinary retention	4 (6.0%)	6 (8.3%)	0.7
Total Complications	12 (17.9%)	8 (11.1%)	0.25

n (%).

Pearson’s Chi-squared test; Fisher’s exact test.

The TP group reported slightly higher rates of acute urinary retention (8.3% vs. 6.0%, p = 0.7). These findings may be partly explained by spinal anesthesia. Bleeding events occurred in both groups and were infrequent and self-limiting. No serious complications (Clavien-Dindo ≥ Grade III) were reported.

## Discussion

This retrospective real-world study compared TR and TP prostate biopsies for diagnostic yield and safety, adding evidence from a region with limited data.

Baseline demographic and clinical characteristics were similar across groups, allowing valid comparisons. However, the transrectal group presented with more suspicious findings on DRE, which could influence cancer detection rates. To address this imbalance, we performed a multivariable analysis adjusting for clinical factors; the results confirmed that the biopsy route was not an independent predictor of csPCa detection (aOR 1.11, p=0.789), demonstrating that the TP approach remains reliable even in populations with fewer palpable tumors.

Both biopsy types detect csPCa similarly, as confirmed by recent randomized trials (6, 7, 13, 14). No independent association between biopsy route and csPCa detection was observed after adjustment. Clinical risk factors—PSA levels, PSA density, and DRE results—were the main determinants of csPCa detection, not biopsy route. Subgroup analysis supports this, as overlapping confidence intervals for odds ratios indicate no clinically significant difference between biopsy routes.

Our cohort identified the TP group as having the best tissue quality, with median core lengths substantially longer than those of the TR group (11.4 mm versus 10.2 mm, p=0.02). The benefit of unrestricted access via a freehand transperineal approach, combined with spinal anesthesia, may limit patient movement, thereby improving the accuracy of sampling.

The identification of csPCa is impacted most by the tumor’s location, with anterior tumors greatly reducing identification potential regardless of biopsy type. Previous studies documented this effect of anterior tumors on the difficulties associated with detecting anterior lesions via standard systematic sampling (15). In our study, the lower detection of anterior lesions despite the technical advantage of the TP route is likely explained using a 12-core systematic template alone, which may not sufficiently sample the anterior zone without additional targeted biopsies.

The safety results of the current study were similar to those reported in other studies (7, 16, 17). TP is widely regarded as having a lower risk of infectious complications (4). This was consistent with our data (1.4% versus 7.5%), although the difference may not have reached statistical significance due to limited sample size. The increased incidence of acute urinary retention in the TP group (8.3% versus 6.0%) may, in part, be attributable to the type of anesthesia used, rather than the biopsy technique.

Recent evidence supports the feasibility and safety of performing transperineal biopsies under local anesthesia, which may further enhance procedural effectiveness (18, 19).

Together, our findings indicate that the consensus is rapidly growing that transperineal (TP) biopsies are a safe and reliable alternative to transrectal (TR) biopsies when performed in a routine clinical setting. Excellent core quality and a low infectious rate further strengthen the case for TP biopsy becoming a standard option - especially in healthcare facilities focused on reducing infectious complications while maintaining diagnostic accuracy.

This study has several noteworthy strengths. Its retrospective design and consecutive patient enrollment minimize selection bias and enhance internal validity. Biopsies were performed by experienced urologists using standardized protocols, ensuring procedural consistency across both routes. Furthermore, the rigorous collection of biopsy-quality metrics provided objective evidence of the technical advantages of the transperineal approach. Importantly, this work contributes clinically relevant data from a tertiary-level Moroccan center—a region underrepresented in the prostate biopsy literature—thereby expanding the geographic diversity of evidence and supporting the external generalizability of comparative TR and TP biopsy performance.

Many limitations exist. The sample size is insufficient to detect differences in infrequent adverse events. Confounding variables arise from differences in anesthetic type between the study groups. Targeted Biopsies were done as part of normal clinical practice, but only Systematic Cores have been examined. This restriction was a deliberate methodological choice to ensure that the template mappings between TR and TP remained consistent, allowing for a pure technical comparison of the two access routes. However, we recognize that this leads to only a partial representation of the absolute diagnostic yield compared to a combined targeted and systematic approach. In addition, the sequential implementation of biopsy techniques may have introduced temporal and learning-curve bias.

## Conclusion

This retrospective comparative study shows that systematic TP biopsy is an alternative to the standard TR approach in clinical practice. A crude analysis first suggested the transrectal route had a higher detection rate, but this was due to more palpable tumors in that group, not technical superiority.

The transperineal method yielded significantly longer biopsy cores. The transperineal approach was also associated with a lower risk of infection than the transrectal approach; however, patients undergoing transperineal biopsy had a slightly increased risk of transient urinary retention.

Taken together, these findings support the growing adoption of the freehand transperineal technique, minimizing infectious risk without compromising diagnostic accuracy. Future multicenter studies incorporating MRI-targeted techniques and larger sample sizes will be essential to further refine biopsy route selection and optimize diagnostic pathways in diverse clinical settings.

## Data Availability

The raw data supporting the conclusions of this article will be made available by the authors, without undue reservation.
